# Gallstone ileus an unusual reason for right iliac fossa pain in Crohn's disease: a case report

**DOI:** 10.4076/1757-1626-2-9285

**Published:** 2009-09-16

**Authors:** Sheharyar Asif Qureshi, Rashaad Gossiel, Mehwish Qureshi

**Affiliations:** 1 Department of Acute Medicine, Queens Medical Centre, Nottingham University Hospitals, Derby Road, Nottingham, UK; 2 Emergency Admissions Unit, Lincoln County Hospital, United Lincolnshire NHS Trust, Greetwell road, Lincoln, UK; 3 Department of Psychiatry, Birmingham Children Hospital, Birmingham and Heartlands NHS Trust, Birmingham, UK

## Abstract

The symptoms of Crohn's disease can vary significantly among afflicted individuals. It is a chronic inflammatory disease, which has many complications involving gastrointestinal and other symptoms. The diagnosis of gallstone ileus is difficult in Crohn's disease and an early diagnosis can reduce mortality. We present a case of 72 year old female with known Crohn's disease with acute right iliac fossa pain. She was investigated with an abdominal CT, giving a definite diagnosis of gallstone ileus.

## Introduction

Crohn's disease is an inflammatory disease, which may effect any part of the digestive system. The main symptoms are abdominal pain, diarrhea, vomiting and weight loss. Abdominal pain may be an initial symptom of Crohn's disease.

Ileocolic Crohn's disease affects the ileum and large intestine accounts of 50 percent of the cases [1]. Gallstone ileus is a rare complication of Crohn's disease. It accounts for 1-4% of all bowel obstructions. Gallstone ileus should always be in the differential diagnosis, when elderly patients are assessed for abdominal pain.

## Case presentation

We present a case of 74-year-old Caucasian women complaining of colicky abdominal pain on admission. She reported acute onset of right iliac fossa pain for 24 hours with associated vomiting but no other associated bowel symptoms. She reported past medical history of hypertension, recent myocardial infarction and Crohn's disease in remission. Her Crohn's was under remission for last ten years without any current treatment. There was no previous history of abdominal surgery. She was non smoker and drank alcohol socially. She was taking no regular medications.

**Figure 1:**
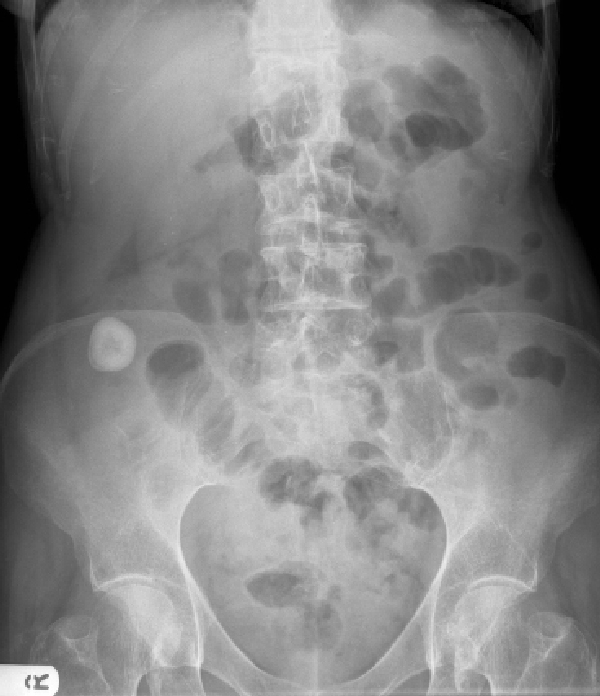
**X-ray of the abdomen showing calcification in right iliac fossa**.

On visual survey, she suffered with no stigmata of a chronic disease. On inspection there were no scars over the abdomen. Her abdominal examination on palpation revealed tenderness in right iliac fossa and loin but no guarding or rigidity. Percussion and auscultation of abdomen was unremarkable. Other systemic examination was normal. Her routine haematology and biochemistry were normal. Her abdominal X-ray revealed a calcification in right iliac fossa (Figure 1).

**Figure 2:**
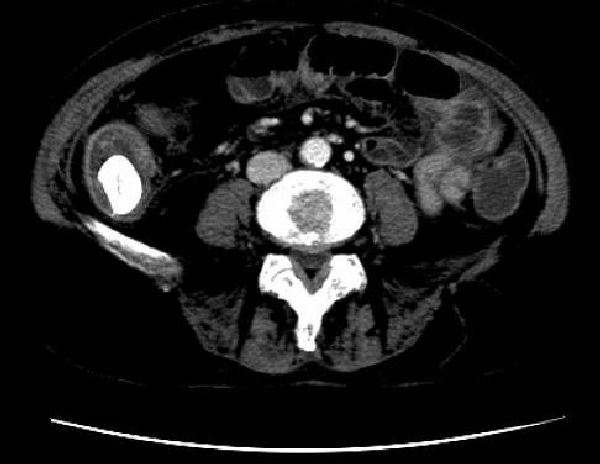
**The CT scan of the abdomen showing gall stone in the small bowel lumen with no signs of bowel obstruction, The bowel wall showing proximal edema to the gall stone**.

The CT scan of the abdomen revealed a gall stone in the small bowel lumen with no signs of bowel obstruction. The bowel wall showed some proximal edema to the gall stone (Figure 2).

She was treated with conservative measures including pain relief and intravenous fluid rehydration. An exploratory supraumbilical laprotomy was performed which revealed impacted gall stone just proximal to the ileocolic junction. She achieved an uneventful recovery and discharged after 5 days of her surgery.

## Conclusion

Crohn's disease is chronic bowel disease, which can lead to several mechanical complications within the intestine. Obstruction typically occurs with strictures and adhesions. The increased prevalence of gall stones in Crohn's disease is thought to be related to depletion of bile salt pool due to terminal ileal disease [2]. Gallstone ileus is a common surgical emergency in older females. It presents during acute medical takes and a delay in achieving diagnosis is not uncommon. If it is left untreated it leads to increased mortality. A high index of suspicion in older patients with intermittent gastrointestinal symptoms should trigger appropriate sensitive investigation like computed tomography of the abdomen.

## Consent

Written informed consent was obtained from the patient for publication of this case report and accompanying images. A copy of the written consent is available for review by the Editor-in-Chief of this journal.

## Competing interests

The authors declare that they have no competing interests.

## Authors' contributions

SQ collected the data and drafted the manuscript with help from MQ. RG provided the supervision for writing the manuscript. All authors read and approved the manuscript.
